# The Human Mind in Its Relations with the Brain and Nervous System

**Published:** 1859-01-01

**Authors:** 


### REVIEWS.

*The Human Mind in its relations with the Brain and Nervous System.* By Daniel Noble, M.D. London, 1858.

A good handy-book on physiological psychology would be a boon to the public, but although the work now lying before us might in bulk, style, and in some respects intention, be considered as a handy-book, it could scarcely be denominated good; for the main object of the author is to set forth certain views on mental physiology which he entertains, but which have not met with general acceptation among physiologists.

Dr. Noble's special opinions on mental physiology are well known among English physiologists, and in a previous number of this Journal (No. XXVIII.) we had occasion to notice their merits as suggestive of inquiry; but now that these opinions are republished in a more elaborate guise, it behoves us rather to examine their demerits, more particularly as the present work is addressed to the amateur and general student as well as to the physiologist and psychologist.

We object to the views which Dr. Noble takes of the position of psychology in scientific research. He writes:—

"In order that a system of analytical psychology should be attained, standing in true scientific relation with our knowledge of the brain, we ought to be able to appreciate the varying phases of consciousness, in watching their outward manifestation, with some of that readiness and accuracy wherewith we can estimate physical conditions. Were this within our power, considerable advances might be made towards a correct and detailed psychology, duly associated with our information concerning the structures within the head. But the inevitable absence of objective standards by which to measure the value of mental facts, materially reduces their comparability among themselves, and with other facts; on this account psychical phenomena do not admit of any natural or perfect system of classification, neither do they allow themselves to be linked-on to physical facts with anything like philosophical exactitude. Yet, of course, scientific induction demands very distinct recognition of the comparable worth of all the circumstances which lead to it."—(pp. 3, 4.)

In this paragraph neither the actual nor the probable position of psychology is, we believe, correctly stated. Recondite and difficult as researches into the nature of psychical phenomena may be, still it does not follow that they will ever stand without the pale of precise and systematic knowledge. Recent progress in psychological research has been such as to warrant the assertion that the conditions upon which it must be followed are becoming clearly known. This is a fundamental condition of scientific research, without which no positive and satisfactory advance can be made. "Psychology holds," as Morell observes, "its proper place in the logical co-ordination of the sciences at large, and will only be *perfected* when all the under-lying data shall have been duly explored and comprehended." Moreover, notwithstanding Dr. Noble's opinion that psychical phenomena do not allow themselves to be linked on to physical facts with anything like philosophical exactitude, yet he subsequently proceeds to give a summary of what he deems to be "the probable, and the *more* than probable, physiology of the nervous system and the various portions of the encephalon, pointing out the correspondence in some detail between it and the more prominent facts of psychology."—(p. 34.)

Again, Dr. Noble writes:—

"In a very early stage of physiological inquiry, the seat of the Soul, or Conscious Principle, was a theme of elaborate and ingenious hypothesis. Hippocrates and Hierophylus place it in the fibres of the brain; Democritus, in the region of the temples; Strabo, in the space between the eyebrows; Epicurus allocated it in the breast; Diogenes, in the left ventricle of the heart; the Stoics, with Chrysippus, in the whole heart; Empedocles placed it in the blood; Plato and Aristotle, with the more elevated schools of philosophy, connected the soul with the whole body; and Galen suggested that each part of the body had its particular soul. In later times, however, conclusions have been attained with regard to the functions of the Encephalon—the structures within the head—which leave no reasonable doubt that the conscious principle has its special seat in that region; conclusions abundantly sustained by evidence from all sources."—(pp. 5, 6.)

Here the soul is confounded with a mode of its manifestation, *consciousness*: a weighty error, we conceive, in psychology.

*Common sensation*, Dr. Noble defines to be "a sense consciousness not limited to any particular organ, but referring itself more or less to the whole frame. . . . This sense resides principally in the skin; it is especially acute at the mucous orifices; it exists, however, in the interior tissues, but in a degree less intense. It is best illustrated by the simple notion of *existence*."—(p. 48.) It would seem that under the designation of common sensation, Dr. Noble includes the special sense of *touch*, and the *common sensibility* of some authors and the *cœnæsthesis* of others; but of this latter form of sensibility more hereafter. Dr. Noble conceives that it is through common sensation that we appreciate the state of the muscles, "experience the *muscular sense*." He then proceeds:—

"This fifth sense (common sensation) is, presumably, awakened through the vesicular extremities—the peripheral expansion—of fibrous filaments. Whether the grey substance and white fibres originating and conducting common sensation be the same as those which subserve the spinal reflex function is uncertain. But this much may be admitted, the communicated impression ascends along the posterior columns of the spinal cord, and attaining gray vesicular centres, produces a consciousness of *common sensation*.

"Physiologists are not agreed as to the identity of these ganglionic structures; they may be expected, however, like the other sensory ganglia, to be somewhere at the base of the encephalon; and I am, myself, disposed to think that the vesicular nuclei within the lateral lobes of the cerebellum—the corpora dentata—constitute the encephalic site of this sense. Many years ago, Foville assigned this function to the aggregate cerebellum; and others, with great plausibility, have advocated this opinion. Dr. Carpenter, however, in his *Human Physiology*, argues against it, on the ground that neither ablation of the organ by operation, nor the destruction of it by disease, have been found to involve the loss of any sensorial capacity. But there may be considerable doubt as to whether, in recorded cases of this kind, the ganglionic extremities of the upper and posterior portion of the spinal cord—the cerebellic termination of the so-called restiform bodies—were actually lost, even though the lobes and their cortical vesicular investment should have disappeared. I doubt if the extension of disease or of experimental excision to structures so closely contiguous to the medulla oblongata as these corpora dentata, would be compatible with the maintenance of functions essential to life; although the removal or destruction of the bulk of the cerebellum, might suggest no such difficulty. Besides, it is notorious that, in the case of animals, movements purely reflex will sometimes be mistaken for those indicative of common sensation. But, probably, the cases already observed with respect to this point, are too few for any decisive conclusion."—(pp. 49—51.)

"The anatomical connexion which exists between the corpora dentata and the posterior columns of the spinal cord, through the corpora restiformia, favours the hypothesis which I have advanced; and various physiological and pathological facts would appear to strengthen it. The experiments of Magendie and Longet show that the slightest touch of the restiform bodies induces violent pain. Hutin relates a case in which the sense of touch was so exalted, that, upon the least contact, intolerable pain and restlessness ensued, with corresponding muscular contractions, resembling those produced by an electric discharge. The patient ultimately died in the most terrific convulsions, prostrate and exhausted. On examination after death, there was found, amongst other changes, atrophy of the cerebellum. 'Its medullary centre, as compared with that of another subject, was a third less in size in either hemisphere. The white substance, which in the normal condition occupies the centre of the corpus rhomboidale, had ceased to exist, so that the fimbriated margins of this portion approached the centre, and only formed a small pyriform, very hard, grayish brown body.'

"Mr. Robert Dunn, of London, a very acute and reflecting practitioner, published a few years ago an interesting and instructive case of tubercle in the brain, wherein there was noticed, amongst other phenomena, imperfect paralysis of the right arm and leg, consisting in failure of common sensation. The patient was a little girl about two years old. 'She could move her arm about,' says Mr. Dunn, 'and could grasp anything firmly enough in her right hand, *when her eyes and attention were directed to it;* but if they were diverted to something else, and the volitional power withdrawn, she would *let the object which she had been holding fall from her hands, and without being conscious of the fact.*' Describing the post-mortem appearances, Mr. Dunn states, 'On making an incision through the lateral lobes of the cerebellum on the left side, I found I had cut through a tubercular deposit, a little to the outer side of the median line (the site of the corpus dentatum), in a state of softened degeneration.'"—(pp. 52—54.)

The experiments of Dr. E. Brown-Séquard seem to afford conclusive evidence against the anatomical arguments of Dr. Noble in favour of the *corpora dentata* of the cerebellum being the ganglionic centres of common sensation. The former gentleman has satisfactorily shown that the posterior columns of the spinal cord are not channels by which sensitive impressions are transmitted to the encephalon. Neither division, nor removal of a portion of the posterior columns is followed by a diminution of sensibility, but, on the contrary, by a marked increase of sensibility in the parts below the portion of the columns incised or removed; and ablation of the restiform bodies is followed by the same results, sensibility being in nowise diminished, but conspicuously increased in the parts below. The conclusions at which Dr. E. Brown-Séquard has arrived from experimental and pathological research, respecting the relations of the posterior columns to sensibility are as follows :—

"The posterior columns of the spinal cord are not, as it has been imagined, a bundle of fibres, from the posterior roots of the spinal nerves going to the encephalon.

"The restiform bodies are not a collection of fibres, chiefly from the sensitive nerves of the various parts of the body, going up to the encephalon, and therefore the cerebellum is not the recipient, through the restiform bodies, of most of the sensitive fibres of the trunk and limbs.

"Deep injuries to the posterior columns of the spinal cord are always followed by a degree of hyperæsthesia greater than after the laying bare of the nervous centres, hyperæsthesia which appears in all parts of the body behind the place injured.

"All the parts of the encephalon which are situated in its posterior or superior side are like the posterior columns of the spinal cord in this respect—that a marked degree of hyperæsthesia always follows a transverse section upon any of them. If a complete transverse section is made upon any part of the restiform bodies, sensibility becomes very much increased in every part of the trunk and limbs. Hyperæsthesia is also, but at a less degree, one of the results of a transverse incision of the cerebellum, in the processus cerebelli ad testes, and in the tubercula quadrigemina.

"The posterior columns of the spinal cord are much less sensitive than they are said to be, and it even seems that the apparent sensibility depends upon the fact, that when they are irritated, the posterior roots, which are sensitive, are also more or less irritated.

"The restiform bodies seem to be deprived of sensibility to mechanical excitation."—(*Lancet, August 7th*, 1858, p. 137.)

Passing over the additional evidence, as well pathological as experimental, which might be adduced to show that the centre of common sensation, or the path of sensitive impressions to the sensorium cannot be in the cerebellum, we would merely add Dr. E. Brown-Séquard's remarks on the case related by Mr. Dunn, and quoted by Dr. Noble. "The case," writes Dr. Brown-Séquard, referring more particularly to the opinion expressed by Dr. Carpenter, that the cerebellum might be the seat of the so-called muscular sense, "certainly seems to be a valuable one; but what can it prove, when we know that movements have remained regular, and, consequently well guided, in many cases in which tubercles, or other morbid products, or various alterations, have existed at the same place where the deposit was found in Mr. Dunn's case."—(*Lancet, August 28th*, 1858, p. 220.)

Dr. Noble appears to regard the *Cœnæsthesis* and *Emotional Sensibility* as one and the same phenomenon :—

"There is yet a sensibility more elevated in the psychical scale than either external sensation or the physical appetites; I refer to that all-pervading sense of substantive existence which German psychologists have named, in some of its phases, *Cœnæsthesis*—general feeling, and sometimes self-feeling (Selbst-Gefühl). It connects itself, apparently, with the peripheral termination of sentient nerves throughout the whole body, but particularly of those supplying the thoracic and abdominal viscera.

"*Emotional* Sensibility, as in the whole of its modifications it may not be inappropriately designated, is experienced in an especial manner about the præcordial region. Its local intensity, indeed, would seem to correspond very much with the prevalence of the vascular system. Under appropriate influences, this sensibility, although more or less general, is always most acutely experienced in the neighbourhod of the large vessels, and most of all about the centre of the circulation; and hence we have the popular as well as poetic localization of 'the feelings' in the *heart*. Yet emotional sensibility is not, like external sensation, of a quasi-physical character; it certainly is not the tactile sensibility of the vascular tubes, which may be affected by many causes influencing the circulation, without there being any resultant effect upon the 'spirits'—another form of popular phraseology which sufficiently indicates, in certain respects, the varying states of this so-called cœnæsthesis." (pp. 60, 61.)

We have already seen that in the term *common sensation* Dr. Noble seems to include the cœnæsthesis properly so called. To make, however, the term cœnæsthesis equivalent to emotional sensibility must have the effect of confusing or rendering valueless the signification of a tolerably well understood word. By cœnæsthesis we have always been accustomed to understand that modification of sensibility which is usually referred to the ganglionic nervous system, and which is familiarly spoken of as common feeling. But, as Feuchtersleben remarks, the word feeling in this term only stands for sensation. Moreover, the term self-feeling differs from the term common feeling in signification, inasmuch as a psychical element is involved in the former.

There is no reason to believe that the relation of the cœnæsthesis to emotion differs from that of any other form of sensation; that is to say, that the psychical relations of the cœnæsthesis are brought about in a different fashion to those of other forms of sensation. This Dr. Noble himself appears to admit, for in his chapter on "The Emotions and their Composition," he writes:—

"If, as I have supposed, the misnamed optic thalami and the corpora striata constitute the ganglionic centres of the several kinds of emotional sensibility, we must in these processes regard them as acted upon from above—from the region of intelligence, the hemispherical ganglia—through the medium of intercommunicating white fibres; just as in cœnæsthetic phenomena dependent upon more physical states, the same centres are supposed to be acted upon from below, through nervous filaments distributed to the organs and structures very generally."—(p. 129.)

Elsewhere, however, throughout the book, Dr. Noble uses the term cœnæsthesis as equivalent to emotional sensibility. Indeed, there is a want of clearness in the mode in which he makes use of the last mentioned term, and also of the term common sensation.

It is not necessary to dwell upon the opinion that the optic thalamus and corpora striata are the ganglionic centres of the so-called emotional sensibility. This conclusion forms a sort of corollary to the opinion that the seat of common sensation is in the corpora dentata of the cerebellum, and it is rendered in a great measure untenable by the evidence in favour of that opinion altogether failing. In addition, the vagueness of Dr. Noble's use of the term "emotional sensibility" vitiates the more specific arguments by which he seeks to determine its seat.

Dr. Noble's remarks on Dr. Carpenter's theory of Unconscious Cerebration are of considerable interest; but does the phrase which Dr. Noble makes use of, "elaboration and perfecting thought *without thought*" rightfully express Dr. Carpenter's idea; or the subsequent phrase, "*involuntary and inattentive thinking*," satisfactorily account for the phenomena sought to be explained in the theory of unconscious cerebration? Dr. Noble writes thus:—

"I conceive that the particular facts, which seem to countenance the theory of unconscious cerebration, will certainly admit of some more obvious and simple interpretation than one which renders it necessary to regard nerve-substance as elaborating and perfecting thought *without thought;* a process, it appears to myself, which would not be altogether unlike the production of melody by a notoriously unmusical instrument without the sensible manifestation of sounds.

"I would here propose to the reader's attention a fundamental consideration bearing upon this question, which is, that the human consciousness, apart from other analyses of which it is susceptible, is traceable under the two forms of *direct* and *reflex*. In the former case ideas are in some sense automatic, and for the most part transient; in the latter they are in their origin to some extent voluntary; or, springing up spontaneously, they become designedly retained in the consciousness, and constitute the material, so to speak, of an objective regard. In solitary musing, when there is no intentioned application of mind to any subject, but rather a passive contentment in our emotional states, consciousness is mostly of the direct character; and, under such circumstances, thoughts and feelings evolve themselves involuntarily—without any sort of effort or purpose. From time to time, however, these mental products are arrested by a reflex act, and the mind voluntarily turns in upon its own thoughts and feelings, thus contemplating not only that which it knows and feels, but its very self at the same time as knowing and feeling.

"Now, although we ordinarily remember facts and mental processes, very much in proportion as they have engaged the attention and a certain reflex consideration at any time, this rule is by no means absolute. Ideas and feelings once experienced may at any time revive in the consciousness, and yet not always be recognised as having previously had existence; particularly when at former periods they have never been subjected, by attention, to a reflex mental process. Undoubtedly, under these latter circumstances, numberless thoughts, and reasonings, and ideas of external occurrences, pass for ever from the consciousness; but this is far from being always the case; again and again will they return, without any systematic identification. And are not most of the phenomena cited by Dr. Carpenter, in support of his theory of unconscious cerebration, explicable by these laws of spontaneous thought, according to which our mental operations are frequently unremembered when repeated. 'Of the thoughts which occur to us suddenly, and which seem to us purely spontaneous, not a few are reminiscences, more or less faithful, of what we have before read, heard, or thought; and consequently they proceed from a preparatory fact which we do not remember' (Balmez).

"And yet this recovered thinking, when attentively regarded, will sometimes seem to have the lucidity and perfection of a special revelation, and may well seem as though it were the product of some unconscious operation of the mental organ. Still, by careful consideration and examination, we shall at times procure demonstration of the contrary. In composition we frequently hit upon an idea, or a word, or the turn of a phrase: it strikes us as a happy thought, and appears to be the spontaneous evolution of our own minds. We afterwards discover, possibly by an accident, that we had heard or read it, yet we had forgotten all about it, and had believed it to be our own. And can we doubt that, in the same way, we sometimes recall our past thinking, deeming it to be new, because we have no conscious remembrance of it? Through ignorance of these laws of thought, or inattention to them, unjust accusations of plagiarism are sometimes made; but 'a writer is not a plagiarist, although he makes ideas his own which have originated with others. But it is often true that man imagines he creates, when he only recollects.'

"In more particular illustration of these phenomena, it may be noted that a book shall be read and soon laid aside; the reader may then pass on to something else, and in a very brief period be unable to render any very clear account of what he has read. Some months afterwards, when the subject of the work becomes a topic of conversation, he is probably surprised that he has derived considerable information from it. How do we explain facts of this kind? Why, in many of such cases, the person situated, as supposed in this illustration, will discover, upon attentive self-examination, that in his passive musings the contents of the book had been in his spontaneous thoughts; and that, under such circumstances, an acquaintance with its subject had been gradually, but still consciously, perfected. This mental process may probably be with some accuracy designated *involuntary and inattentive thinking,* but not with justice an *unconscious action of the brain.* I am decidedly of opinion, myself, that the explanation now offered of these well-known phenomena will more or less cover all the psychical processes that have been cited to establish a doctrine of Unconscious Cerebration." (pp. 94—99.)

In a chapter on the "Physiological Potency of Ideas," Dr. Noble illustrates his subject by several singularly interesting cases; and in a subsequent chapter he demurs to pleasure and pain being made the last analysis of emotion. He writes:—

"The inward feelings called forth, as emotion, by the agency of thought, may, of course, be pleasurable or painful; but any account which represents the 'Emotions' as merely the pleasure or the pain which accompanies certain intellectual states, constitutes a very incomplete description. Yet the late Mr. James Mill, the Rev. Sydney Smith, and many others, would seem to reduce them to so very simple a character; although in practical and extended discussion, other views become implied, in disregard of strict logical consistency. Benevolence, considered in this way, becomes the pleasure experienced in contemplation of the happiness of others, and the pain at witnessing their misery; and fear, again, as the pain that ensues upon the expectation of calamity; an analysis being thus attainable with all our emotional states — passions, affections, and sentiments alike.

"Now, I think it will be conceded, upon reflection, that we must admit the specifically distinct character of our varying states of consciousness, as recognised in Hope, Fear, Grief, Pride, Vanity, Love, and other such inward experiences. 'Sentiment,' says Rosmini, 'has various states, pleasurable and painful, with gradation and *variety* of pleasure, and with gradation and *variety* of pain.' And, somewhat more explicitly, in another place, he observes:—'The sentiments correspond with *orders of reflection;* so that there are as many orders of feeling (pleasurable or painful) to be noted as there are orders of reflection exercised by man, and the number of these is indefinite.'" (pp. 130—132.)

In this opinion of Dr. Noble's many psychologists will concur.

We have confined our attention to the portions of this work in which Dr. Noble's opinions are most prominent, and we have thought it our duty to state in what respect we differ from him. This course was more necessary in regard to a work which will probably find its way largely into the hands of amateurs and general students, by whom Dr. Noble's opinions will most likely be received, from the authority of his name, as of higher value than he himself would desire; for the learner is not always apt in distinguishing hypothesis from ascertained fact, even when, as in the case of Dr. Noble's hypotheses, he is duly cautioned by the author.

Of the whole of Dr. Noble's work, it may be said that it is most interesting to read, and that it contains no small amount of information very agreeably set forth.

